# Olfactory Malformations in Mendelian Disorders of the Epigenetic Machinery

**DOI:** 10.3389/fcell.2020.00710

**Published:** 2020-08-04

**Authors:** Sebastiano Aleo, Claudia Cinnante, Sabrina Avignone, Elisabetta Prada, Giulietta Scuvera, Paola Francesca Ajmone, Angelo Selicorni, Maria Antonella Costantino, Fabio Triulzi, Paola Marchisio, Cristina Gervasini, Donatella Milani

**Affiliations:** ^1^Pediatric Highly Intensive Care Unit, Fondazione IRCCS Ca’ Granda Ospedale Maggiore Policlinico, Milan, Italy; ^2^Fondazione IRCCS Ca’ Granda Ospedale Maggiore Policlinico, Neuroradiology Unit, Università degli Studi di Milano, Milan, Italy; ^3^Child and Adolescent Neuropsychiatric Service (UONPIA), Fondazione IRCCS Ca’ Granda Ospedale Maggiore Policlinico, Milan, Italy; ^4^Unit of Pediatric, Presidio S. Fermo, ASST Lariana, Como, Italy; ^5^Department of Pathophysiology and Transplantation, Università degli Studi di Milano, Milan, Italy; ^6^Division of Medical Genetics, Department of Health Sciences, Università degli Studi di Milano, Milan, Italy

**Keywords:** epigenetics, neuroimaging, nervous system diseases/genetics, olfactory bulb, rare diseases, mendelian disorders of the epigenetic machinery

## Abstract

Usually overlooked by physicians, olfactory abnormalities are not uncommon. Olfactory malformations have recently been reported in an emerging group of genetic disorders called Mendelian Disorders of the Epigenetic Machinery (MDEM). This study aims to determine the prevalence of olfactory malformations in a heterogeneous group of subjects with MDEM. We reviewed the clinical data of 35 patients, 20 females and 15 males, with a mean age of 9.52 years (SD 4.99). All patients had a MDEM and an already available high-resolution brain MRI scan. Two experienced neuroradiologists reviewed the MR images, noting abnormalities and classifying olfactory malformations. Main findings included Corpus Callosum, Cerebellar vermis, and olfactory defects. The latter were found in 11/35 cases (31.4%), of which 7/11 had Rubinstein-Taybi syndrome (RSTS), 2/11 had CHARGE syndrome, 1/11 had Kleefstra syndrome (KLFS), and 1/11 had Weaver syndrome (WVS). The irregularities mainly concerned the olfactory bulbs and were bilateral in 9/11 patients. With over 30% of our sample having an olfactory malformation, this study reveals a possible new diagnostic marker for MDEM and links the epigenetic machinery to the development of the olfactory bulbs.

## Introduction

As we breathe, millions of airborne odorants get captured by appropriate receptors embedded in the cellular plasma membrane of sensory neurons located deep inside the nasal cavity thus initiating a complex biochemical response that ultimately leads to the perception of odors. The olfactory epithelium, a relatively simple tissue home to the primary sensing cell of the system, and the olfactory bulbs (OBs), true neocerebral extensions, are two key structures to human and mammalian olfaction.

Although usually overlooked by physicians, olfactory abnormalities are not uncommon, with many conditions, mainly non-genetic, causing some disruption to the system. To the best of our knowledge, among these conditions are only a few complex genetic syndromes such as Kallman syndrome 1 (KS1, #308700), and CHARGE syndrome (#214800). The latter has recently been classified among an emerging group of extremely rare genetic disorders, the Mendelian Disorders of the Epigenetic Machinery (MDEM) (Fahrner and Bjornsson, 2019). Despite being a relatively young group, more than 80 conditions have already been identified. Common features are intellectual disability, abnormal growth, and brain and limb malformations. Disease severity and clinical presentation, however, vary widely.

Although a clear and widely accepted definition of the term does not exist, epigenetics studies inheritable changes of the genetic expression that do not involve actual changes in the DNA sequence. The epigenetic machinery regulates a cell’s gene expression primarily through DNA methylation, histone modifications, and nucleosome positioning. Interestingly, long non-coding RNAs have been recently shown to interact and regulate chromatin-modifying complexes, thus adding an extra layer of complexity to this already intricate area of molecular biology. Various molecules make up the epigenetic machinery, and can be classified into four groups: (a) writer proteins, molecules whose primary role is to add chemical groups to DNA or histones; (b) reader enzymes, able to identify changes made by writer proteins, and recruit big protein complexes which, in turn, alter the chromatin structure, suppressing or favoring gene expression of that particular area of DNA; (c) eraser enzymes, which remove the added chemical groups, thus restoring the original expression pattern; and (d) remodeler proteins, a group of ATP-dependent enzymes able to alter protein-DNA interaction by modifying nucleosome positioning. Early disruption in one of these mechanisms can determine inappropriate gene expression or suppression, and the possible onset of a MDEM (Fahrner and Bjornsson, 2014).

As these conditions suggest, epigenetics plays an essential role in the development of the central nervous system (CNS), and it is, therefore, feasible that olfactory malformations are present in other MDEM besides CHARGE syndrome.

Basic olfactory anatomy has remained unchanged across 500 million years of vertebrate evolution (Hoover, 2010) and consists of two independent components: the main olfactory system for detecting odorants and the accessory olfactory system.

Basic olfactory circuitry begins in the nasal cavity where the receptors of olfactory sensory neurons (OSNs) detect odor signals. Following olfactory transduction, the information is relayed through action potentials to the OBs, where it is then processed. The OB, a complex twin ovoid structure located just above the cribriform plate in the anterior cranial fossa, is home to many different types of neurons, mainly interneurons. The latter modulate the activity of mitral and tufted cells, second-order neurons, with the important role of conveying afferent odor information to various cortical regions via the olfactory tract.

The olfactory epithelium (OE) and the OB start independent, but almost simultaneous development early in the embryo with the OE arising from the olfactory placode, and the OB emerging from a predetermined region of the rostral telencephalon (López-Mascaraque and de Castro, 2002; Sarnat and Yu, 2016). The development of the OE and the OB are not independent processes as is demonstrated by the detachment of an accumulation of axons and cells, termed “migratory mass,” from the olfactory placode. The detached cells navigate to the rostral portion of the telencephalon contributing to the formation of the olfactory nerve and inducing the macroscopic evagination of the OB (Valverde et al., 1992).

When searching for brain malformations, magnetic resonance imaging (MRI) is usually the method of choice. By outputting detailed images, routine MRI can be used to extensively analyze the brain including olfactory structures, such as the OBs, the olfactory tract, the olfactory cortex, and the olfactory sulci. These structures are well assessed in conditions known to have abnormal olfactory development, such as Kallmann syndrome and CHARGE syndrome. Brain MRI of these syndromes usually demonstrates absence or hypoplasia of the OB and marked reduction in the olfactory sulcus depth. A qualitative or quantitative assessment can be used to describe these abnormalities. In conditions where these neuroradiological alterations are not typical, the olfactory structures are seemingly overlooked, with consequently fewer reports of malformations compared to other brain structures.

Brain malformations are probably present in most MDEM, but not all these conditions are assessed with brain MRI. Considering the need for sedation in most children, intellectual disability is not always severe enough to require brain imaging evaluation. Rubinstein-Taybi syndrome (RSTS1 *#180849*, RSTS2 *#613684*) is one of the MDEM most frequently assessed with brain MRI.

This work is aimed at determining the prevalence of olfactory abnormalities in a heterogeneous group of patients with MDEM and at improving our knowledge on the neuroradiological spectrum of these complex conditions. Comparison with a control group will help add specificity to our findings.

## Materials and Methods

Subjects included in the study had a clinically and molecularly proved MDEM and an available brain MRI scan. All patients are followed at our pediatric center in Milan (Italy).

From the participants’ medical records we collected sex, age, clinical diagnosis, molecular diagnosis, and MR images. The data was then used to construct a database (available in Supplementary Material).

Available MR images had been performed on a 1.5 T unit or higher and were independently reviewed by two experienced neuroradiologists. Blinding was not feasible, and both experts were aware of the clinical diagnosis. Brain malformations were listed as being present or absent, and olfactory dysmorphisms were further classified into hypoplasia or aplasia, and into left, right, or bilateral.

To better interpret our results, brain MR images of a control group were also reviewed. These patients were randomly extracted from our Pediatric Highly Intensive Care Unit’s database after excluding subjects with possible MDEM, and filtering for neurological syndromes and available brain MRI scans. Both neuroradiologists were rendered unaware of the different underlying conditions. Olfactory abnormalities were the only studied feature on brain MR images for the control group. We used Fisher’s exact test to compare OB abnormality rates between the two groups. A *P*-value of less than 0.05 was considered to indicate statistical significance. Statistical analysis was performed using R version 4.0.2 (2020-06-22).

Inclusion/exclusion criteria and study methodology were constructed before data collection assessment.

### Ethics Statement

This study was approved by the local Ethical Committee “Milano Area 2.” All patients have been provided with written informed consent to the publication of this study’s results.

### Participants

Of the 35 patients included in the MDEM group, 20 were females and 15 were males, with a mean age of 9.52 years (SD 4.99; Table 1), at the time of writing. We identified 11 different conditions in all: 14/35 patients had Rubinstein-Taybi syndrome, accounting for 40% of the sample; 5/35 children had Sotos syndrome (SS1 *#117550*); 4/35 children had Kleefstra syndrome (KLEFS *#617768*); 3/35 had Coffin-Siris syndrome (CSS1 *#135900*); 2/35 had Wiedemann-Steiner syndrome (WDSTS *#605130*); 2/35 had CHARGE syndrome; 2/35 patients had Smith-Magenis syndrome (SMS *#182290*). Also included in the study were three patients with, respectively, Kabuki syndrome (KMS1 *#147920*), Floating-Harbor syndrome (FLHS *#136140*), and Weaver syndrome (WVS *#277590*). The corresponding molecular features are presented in Table 1. Available accession numbers can be found in the provided Supplementary Material.

**TABLE 1:** Characteristics and diagnosis of recruited subjects.

ID	Gonadal sex	Age (years)	Clinical diagnosis	Molecular diagnosis
1	F	5,5	CHARGE	*CHD7*: c.8077-1G > A
2	M	2,8	CHARGE	*CHD7*: c.6492insA; p.(Val2315Serfs*11)
3	M	3,8	CSS	*ARID1B*: c.5049del; p.(Val1684Serfs*8)
4	F	7,3	CSS	*ARID1B*: c.6164G > A; p.(Trp2055*)
5	F	7,8	CSS	*ARID1B*: c.382G > T; p.(Glu1276*)
6	F	6,4	FLHS	*SRCAP*: c.7394delC; p.(Pro2455Glufs*10)
7	F	8,9	KBS	*KMT2D*: c.16295G > A; p.(Arg5432Gln)
8	M	14	KLFS	*EHMT1*: c.3202T > A; p.(Cys1068Ser)
9	F	2,8	KLFS	arr 9p21.3del; 9q34.3del (*EHMT1*-)
10	M	3,8	KLFS	arr 9q34.3del (*EHMT1*-)
11	M	4	KLFS	arr 9q34.3del (*EHMT1*-)
12	M	14,5	RSTS	*CREBBP*: c.G4435T; p.(Gly1479*)
13	M	10,8	RSTS	*CREBBP*: c.1915_1916dupGA; p.(Asp639Glufs*17)
14	M	25,8	RSTS	*EP300*: c.4640dupA; p.(Asn1547Lysfs*3)
15	F	5,5	RSTS	arr 16p13.3del (*CREBB*P-)
16	F	19	RSTS	*CREBBP*: c.2616dupG; p.(Thr873Aspfs*97)
17	F	12,5	RSTS	*CREBBP*: c.4492C > T; p.(Arg1498*)
18	F	15,3	RSTS	*CREBBP*: c.1570dupC; p.(Leu524Profs*2)
19	F	7,3	RSTS	*CREBBP*: c.4650_4654delAGAGA; p.(Glu1551Hisfs*2)
20	M	10,1	RSTS	*CREBBP:* c.(5421_5648)_(8131_?)del (MLPA ex29-31del)
21	F	3,7	RSTS	*CREBBP*: c.(4742_4856):(8131_?)del (MLPA ex24-31del)
22	F	12,7	RSTS	*CREBBP*: c.3610-2A (>G
23	F	13,9	RSTS	arr 3p12.3p14.1dup
24	M	7,6	RSTS	*CREBBP* c.3524A > G; p.(Tyr1175Cys)
25	F	10,7	RSTS	*CREBBP*: c.1483C > T; p.(Glu495*)
26	F	17	SMS	*RAI1*: c.832_834delCAG; p.(Gln278del)
27	F	12,8	SMS	FISH del(17) (p11.2p11.2) (RAI1-)
28	F	11,2	SS	*NSD1*: c.4792C > T; p.(Gln1598*)
29	M	9,5	SS	*NSD1*: c.1527delT; p.(Ser510Valfs*2)
30	M	6,7	SS	*NSD1*: c.1810C > T; p.(Arg604*)
31	F	7	SS	*NSD1*: c.3958C > T; p.(Arg1320*)
32	M	9	SS	*NSD1*: c.1107delT; p.(Phe369Leufs*50)
33	M	7,8	WVS	*EZH2*: c.2050C > T; p.(Arg684Cys)
34	M	8,7	WSS	*KMT2A*: c.3294G > A; p.(Trp1098*)
35	F	7,1	WSS	*KMT2A*: c.3461G > A; p.(Arg1154Gln)

*CSS, Coffin-Siris syndrome; FLHS, floating harbor syndrome; KBS, Kabuki syndrome; KLFS, Kleefstra syndrome; RSTS, Rubinstein-Taybi syndrome; SMS, Smith-Magenis syndrome; SS, Sotos syndrome; WVS, Weaver syndrome; WSS, Wiedemann-Steiner syndrome. Reported sequence variants were described following den Dunnen et al.’s (2016) HGVS recommendations for the description of sequence variants: 2016 update.*

On evaluating available MRI data, 18/35 patients had only brain MR images, while 17/35 children had also a whole-spine MR image. These whole-spine MR images were obtained from 12/17 patients with RSTS, 2/17 with CHARGE syndrome, 2/17 with KLFS, and 1/17 with KMS.

The control group was made up by 35 patients, 18 males and 17 females, and included a wide variety of conditions. Not all subjects had a clinically and molecularly proven disorder (see Data Sheet in Supplementary Material).

## Results

The most common feature upon evaluating brain MRI scans was a dysmorphic corpus callosum (CC), found in 18/35 patients (51.4%). In 6/18 patients this was the only brain abnormality present.

Along with the CC, another common feature was cerebellar vermis dysmorphisms (extra rotation or hypoplasia), in 10/35 children (28.5%).

Other abnormalities included a dysmorphic brain stem in 5/35 patients, Arnold-Chiari malformation in 3/35, optic nerve abnormalities in 3/35 and hippocampus malformations in 2/35.

The 17 available whole spine MRI records showed a medullary cone located at or below L2-L3 lumbar vertebrae space in 7/35 patients (41.2%). All 7 patients had RSTS. Table 2 summarizes our findings by MDEM syndrome and localization.

**TABLE 2:** Number of identified malformations on Brain MRI scans.

Clinical syndromes	CHARGE	CSS	FLHS	KBS	KLFS	RSTS	SMS	SS	WVS	WSS	Total
*n*^†^	2	3	1	1	4	14	2	5	1	2	35
Corpus callosum	2	3	1	0	2	10	0	3	1	0	22
Olfactory bulbs	2	0	0	0	1	7	0	0	1	0	11
Olfactory tracts	2	0	0	0	0	0	0	0	0	0	1
Cerebellum	2	0	0	0	1	5	0	0	0	0	8
Brain stem	2	1	0	0	1	2	0	0	0	0	6
Fornix	2	0	0	0	0	0	0	0	0	0	1
Optical tracts	1	0	0	0	0	2	0	0	0	0	3
Enlarged ventricules	1	1	0	0	3	3	1	2	0	0	10
Anterior commissure	0	0	0	0	0	2	0	0	1	0	3
Hyppocampus	0	0	0	0	0	2	0	0	0	0	2
ACM^‡^	0	2	0	1	0	1	0	0	0	0	4

*CSS, Coffin-Siris syndrome; FLHS, floating harbor syndrome; KBS, Kabuki syndrome; KLFS, Kleefstra syndrome; RSTS, Rubinstein-Taybi syndrome; SMS, Smith-Magenis syndrome; SS, Sotos syndrome; WVS, Weaver syndrome; WSS, Wiedemann-Steiner syndrome. ^†^Number of patients. ^‡^Arnold Chiari Malformation.*

**FIGURE 1:**
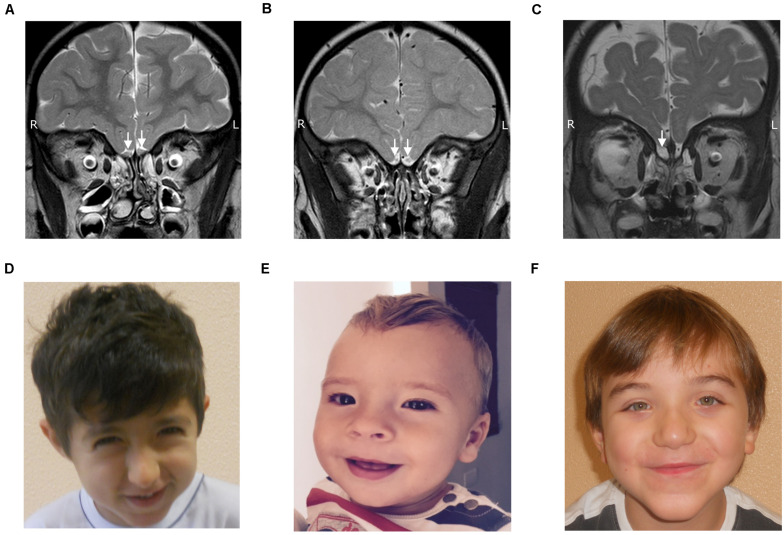
TSE T2 weighed coronal images showing normal olfactory bulbs **(A)**, olfactory bulb hypoplasia **(B)** and olfactory bulb aplasia **(C)**, along with facial features of RSTS **(D)**, KLFS **(E)** and WVS **(F)**.

Olfactory abnormalities were among the most common malformations and were depicted in 11/35 patients (31.4%), six females and five males. More specifically, they were present in 7/14 children with RSTS (50%), 2/2 children with CHARGE syndrome, 1/4 with KLFS and 1/1 with WVS (see Figure 1). The findings in RSTS thus accounted for 63% (7/11) of the olfactory abnormalities present in our series. Irregularities mainly concerned the OBs, while the other olfactory structures were more or less spared. The OBs were involved in all 11 cases, bilaterally in 9/11 patients and unilaterally in 2/11 patients. The right OB was hypoplastic in 6/11 cases (54%) and aplastic in 5/11 (46%). The left OB, on the other hand, was abnormal in 9/11 cases, with hypoplasia in 8/9 patients (88%), and aplasia in 1/9 (12%). OB defects per subject are shown in Table 3.

**TABLE 3:** Classifying OB^†^ malformations.

ID	Gonadal sex	Left OB	Right OB
1	F	1	2
2	M	2	2
11	M	1	1
13	M	0	2
14	M	1	1
15	F	1	1
18	F	1	1
22	F	0	2
23	F	1	1
25	F	1	2
33	M	1	1

*Olfactory bulb (OB) abnormalities classified by side and grade. The number “0” represents a normal OB, while “1” and “2,” respectively, indicate hypoplasia and aplasia.*

Along with OB malformations, the only other disruption noted in the olfactory system was that of the olfactory tracts in two patients with CHARGE syndrome; none of the other nine patients displayed additional olfactory irregularities.

Out of the control group, 2/35 subjects (5.7%), one with neurofibromatosis type 1 (NF1, *#162200*) and one with Gorlin-Goltz syndrome (GGS, *#109400*), were found to harbor an OB abnormality. While the former had a bilateral OB hypoplasia, the patient with GGS had a right OB hypoplasia and a left OB aplasia (see Data Sheet in Supplementary Material).

The different number of OB abnormalities found when comparing the two groups was statistically significant (*P* = 0.0118).

## Discussion

Proper control of gene expression is necessary for the normal development of brain structures and function. Therefore, when genetic disorders disrupt epigenetic machinery molecules, brain malformations are a near-constant. This seems especially true for median structures of the brain, such as the corpus callosum and the cerebellar vermis as reported by different studies (Schaefer et al., 1997; Schrier et al., 2012; Willemsen et al., 2012; Maya et al., 2014; Ajmone et al., 2018). As more and more studies on the neurological aspects of the MDEM emerge, reports on other brain malformations are revealed. Searching the literature for OB hypoplasia/aplasia in these conditions unveiled a large number of studies involving CHARGE syndrome (Lin et al., 1990; Harvey et al., 1991; Pinto et al., 2005), but only a couple of human reports on RSTS and KLFS (Ajmone et al., 2018; Ciaccio et al., 2019).

Although highly unlikely, OB hypoplasia/aplasia may also be a common finding among the general population. A recent review of a public brain MRI database with 1,113 participants described agenesis of the OBs in five otherwise healthy women. Interestingly all had a normal or above-average olfactory function. The authors conclude that olfaction without apparent OBs is evident in 0.6% of women and 4.25% of left-handed women (Weiss et al., 2019). Our series presented a much higher prevalence of OB malformations and an equal distribution between males and females. These results hint to an important role of the epigenetic machinery in controlling the development of the olfactory system. It is necessary to point out, however, that an over-representation of patients with RSTS may constitute an important bias in our study, altering the real prevalence of OB abnormalities in MDEM.

Smaller or absent OBs could arise from specific defects in neural differentiation and/or migration. These complex processes, by which proper brain development depends, are governed by epigenetics (Hsieh and Gage, 2005; Sweatt, 2013). Cell migration, for example, relies on the ability to detect and translate external cues into intracellular signals by which different pathways and transcription factors are then activated or suppressed (Franz et al., 2002; Buchsbaum and Cappello, 2019). It goes without saying that precise timing and correct gene expression/suppression are required for a cell to be able to migrate.

Migration and differentiation may seem independent processes, however, a premature failure in differentiation will most probably give rise to a perturbed migration by lack of required molecules.

Brain defects in which the inability of neurons to migrate plays a central role are known as Neuronal Migration Disorders. Some of these defects, such as Cerebellar hypoplasia, CC agenesis, Dandy-Walker malformation, overlap with those reported in MDEM. This may suggest that neuronal migration defects are what determine brain malformations in these syndromes. The presence of dysgenesis in olfactory structures, found in 30% of our patients, is consistent with this hypothesis. In zebrafish, the region which gives rise to the olfactory placodes is, in fact, characterized by little cell division and is formed primarily through cellular migration (Miyasaka et al., 2013). Multiple cell types originate within this region but then migrate, following olfactory sensory neuron’s (OSN) axons, toward the nascent OBs (Valverde et al., 1992).

The presence of OB defects in CHARGE syndrome, RSTS, KLFS, and WVS, point to a connection between the specific gene defects of these syndromes and olfactory development. Establishing a definite relation is beyond the scope of this study, however, genes involved in these four conditions all seem to be linked, some way or another, to neuronal differentiation, maturation, and migration. *De novo* mutations in the gene encoding for Chromodomain Helicase DNA binding protein 7 (*CHD7, #608892*), for example, are a major cause of CHARGE syndrome. This enzyme has been found to regulate several mouse genes involved in neural cell migration and guidance, including semaphorins, such as Sema3a, and ephrin receptors (Schulz et al., 2014). Similarly, cAMP-response element-binding protein (*CREBBP*, *#600140*) gene and of E1A Binding Protein P300 (*EP300, #602700)* gene, both involved in RSTS, can control the expression of numerous genes important for cell differentiation and maturation (Goodman and Smolik, 2000; Zimmermann et al., 2007). Reduced levels of functioning protein lead to disrupted gene expression/suppression and dysregulated differentiation and maturation of different classes of neurons during development (Wang et al., 2010; del Blanco et al., 2019; Schoof et al., 2019). Direct evidence of aberrant neuronal migration has also been reported in WVS. In 2013 Tatton-Brown et al. first described a case of pachy- and polymicrogyria in a WVS patient with a truncating variant in enhancer of zeste homolog 2 (*EZH2*, *#601573*) (Tatton-Brown et al., 2013). *EZH2* has been shown to be highly expressed in embryonic tissues and proliferating cells and is important in maintaining the number of neuronal precursors which manage to differentiate into mature neurons (Schuettengruber et al., 2007; Pereira et al., 2010; Marei et al., 2012). Pachy- and polymicrogyria are both considered malformations of cortical development due to defects in neuronal migration and/or cortical organization. Pachygyria may develop when large number of neurons fail to reach the cortex, while polymicrogyria is thought to arise from a disruption in a later stage of migration (Francis et al., 2006).

In contrast to *CHD7*, *CREBBP/EP300*, and *EZH2*, euchromatic histone lysine methyltransferase 1 (*EHMT1, #607001*), involved in KLFS has not yet been directly associated with neural differentiation and migration. This methyltransferase is, however, required for normal neuronal electrophysiological activity. By establishing synaptic connections *EHMT1* may contribute significantly to neuronal survival (Martens et al., 2016).

NF1 and GGS were the two conditions associated with OB abnormalities within the control group. GGS is a rare autosomal dominant disorder characterized by basal cell carcinomas, dental and osseous defects, and neurological and sex organ abnormalities. Clinical presentation is extremely variable, with more than 100 minor criteria described (Jawa et al., 2009). Among possible presentations, absent OBs and hypogonadotropic hypogonadism have been recently reported (Barraud et al., 2020). Unlike GGS, to our knowledge, no olfactory malformation has ever been described in NF1 patients. Although the presence of OB defects in these two control group patients may question the specificity and diagnostic reliability of such a finding in possible MDEM syndromes, we are inclined to consider both occurrences rare and of less significance in a diagnostic context.

## Conclusion

In conclusion, major efforts have been recently made to better characterize MDEM. Brain MRI scanning has commonly revealed brain malformations, which vary in type and severity. Our study focused on evaluating the presence of olfactory abnormalities in available brain MRIs of patients with MDEM. Olfactory structure anomalies were noted in RSTS, CHARGE syndrome, KLFS, and WVS, although due to a relatively small sample size these results may be inaccurate. OB malformations and other abnormalities commonly found in these syndromes, such as cerebellar hypoplasia and CC agenesis, may find a common cause in neuronal migration defects. This is supported by the fact that genetic defects found in RSTS, CHARGE syndrome, KLFS and WVS are all linked, in some way or another, to neuronal differentiation, maturation, and migration.

Congenital anomalies in olfactory structures are already an important diagnostic feature in CHARGE syndrome. Our findings in MDEM coupled with a low rate of OB malformations in other neurological syndromes, highlights the possibility for these abnormalities to become soft markers of RSTS, KLFS, and WVS. Assessment of olfactory structures thus becomes crucial when evaluating a brain MRI scan. Although we acknowledge that the relatively small sample size is an important limitation to the statistical significance of our study, MDEM are extremely rare, and bigger cohorts are difficult to assemble. Future studies will help assess the real prevalence of olfactory defects and their real use as markers in these conditions.

## Data Availability Statement

All datasets presented in this study are included in the article/Supplementary Material.

## Ethics Statement

The studies involving human participants were reviewed and approved by Ethical Committee Milano Area 2. Written informed consent to participate in this study was provided by the participants’ legal guardian/next of kin. Written informed consent was obtained from the minor(s)’ legal guardian/next of kin for the publication of any potentially identifiable images or data included in this article.

## Author Contributions

SAl designed and wrote the manuscript in consultation with DM and CC. SAv and CG analyzed the data and provided critical feedback. GS, EP, AS, and PA contributed to the design and implementation of the research. FT, MC and PM helped supervise the project. All authors contributed to the article and approved the submitted version.

## Conflict of Interest

The authors declare that the research was conducted in the absence of any commercial or financial relationships that could be construed as a potential conflict of interest.
